# Trench Fever. Report of Commission. Medical Research Committee, American Red Cross

**Published:** 1919

**Authors:** 


**Trench Fever. Report of Commission. Medical Research Committee, American Red Cross.** Pp. vii., 446. Oxford Medical Publications. 1918. Price 21s. net.

It is impossible for any medical man who has spent any length of time on the Western Front not to be interested in this book. Trench Fever was as baffling as any disease could be, and in spite of a mortality of zero, was causing more wastage from the fighting-line than any other disease, with the exception of the ever-present I.C.T. The reviewer well remembers its first appearance in the battalion of which he was in medical charge: how it struck an entire platoon who were sleeping in the cellar of a ruined house in the "support line," and how he was utterly unable, after blood-cultures, etc., for the typhoid group had proved negative, to persuade the A.D.M.S. that anything serious ailed the men, who ran temperatures of 99° odd every evening, with an occasional jump to 101° or so. This was in the summer of 1915. Early in 1916 McNee and others published experiments, in which he transmitted the disease from Trench Fever patients to healthy individuals by direct blood transfusion. For some mysterious reason no one took much notice, nor were his results confirmed, in spite of the fact that by this time, the spring of 1916, the disease reached quite alarming proportions. A certain number of spasmodic ideas were projected, it is true, and there arose one party who contended that Trench Fever was a *louse-borne* diease, while another party contended with equal fervour on equally insufficient evidence that it was not. On America joining the Allies the American Medical Service realised the importance of the matter. Accordingly, with the cordial co-operation of the R.A.M.C., they set up a commission and started work early in 1918. This book is a report of their labours. It tells of their methods and their findings; how they confirmed McNee's experiments made two years previously and proved incidentally that the different clinical types which we all had learned to recognise were one and the same disease; how the disease is undoubtedly *louse-borne;* and how the organism is a filtrable virus, and has nothing to do with the enteric group. It tells of the precautions they took to prevent error and faulty conclusions. And all this the American Commission did in two months. The report is a monument to organised effort or team-work as opposed to sporadic endeavour. Moreover, the text, written by Major Strong, M.R.C., the head of the Commission, will not jar on the purist, to whom so many American medical publications are anathema.

